# Heterodimeric protein complex identification by naïve Bayes classifiers

**DOI:** 10.1186/1471-2105-14-347

**Published:** 2013-12-03

**Authors:** Osamu Maruyama

**Affiliations:** 1 Institute of Mathematics for Industry, Kyushu University, Fukuoka, Japan

**Keywords:** Heterodimeric protein complex, Supervised learning, Naïve Bayes classifier, Protein-protein interaction

## Abstract

**Background:**

Protein complexes are basic cellular entities that carry out the functions of their components. It can be found that in databases of protein complexes of yeast like CYC2008, the major type of known protein complexes is heterodimeric complexes. Although a number of methods for trying to predict sets of proteins that form arbitrary types of protein complexes simultaneously have been proposed, it can be found that they often fail to predict heterodimeric complexes.

**Results:**

In this paper, we have designed several features characterizing heterodimeric protein complexes based on genomic data sets, and proposed a supervised-learning method for the prediction of heterodimeric protein complexes. This method learns the parameters of the features, which are embedded in the naïve Bayes classifier. The log-likelihood ratio derived from the naïve Bayes classifier with the parameter values obtained by maximum likelihood estimation gives the score of a given pair of proteins to predict whether the pair is a heterodimeric complex or not. A five-fold cross-validation shows good performance on yeast. The trained classifiers also show higher predictability than various existing algorithms on yeast data sets with approximate and exact matching criteria.

**Conclusions:**

Heterodimeric protein complex prediction is a rather harder problem than heteromeric protein complex prediction because heterodimeric protein complex is topologically simpler. However, it turns out that by designing features specialized for heterodimeric protein complexes, predictability of them can be improved. Thus, the design of more sophisticate features for heterodimeric protein complexes as well as the accumulation of more accurate and useful genome-wide data sets will lead to higher predictability of heterodimeric protein complexes. Our tool can be downloaded from http://imi.kyushu-u.ac.jp/~om/.

## Background

Protein complexes are basic cellular entities that carry out the functions of their components. Those functions are performed in various biological processes in a cell, including transcription, signal transduction, and development. Therefore, it is useful to identify protein complexes in order to elucidate various complicated mechanisms in a cell.

There exist a few databases of protein complexes of yeast. One of them is MIPS protein complex catalog [1], a comprehensive catalog of manually curated protein complexes of yeast. It contains 217 complexes, excluding complexes derived from high-throughput experimental data sets [2-4]. An up-to-date database of curated protein complexes of yeast is CYC2008 [5], which contains manually curated 408 heteromeric protein complexes.

The major type of protein complexes in these databases is heterodimeric complexes, that is, protein complexes composed of two different proteins. MIPS catalog and CYC2008 have 64 (29%) and 172 (42%) heterodimeric complexes, respectively.

A number of unsupervised learning methods for predicting arbitrary types of protein complexes simultaneously have been proposed, for example, MCL [6], RRW [7], NWE [8], PPSampler [9], RNSC [10], MCODE [11], DPClus [12], CMC [13], and COACH [14]. It is reported that they achieve good performance for protein complexes of size three or more (see, for example, [15-17]). However, it can be found that those existing tools can not give high performance for heterodimeric complexes [9]. The best known F-measure score is only 0.316, which is achieved by PPSampler (Details will be given in the result section).

A fundamental reason of the drawback is that a score function for a predicted complex is often designed based on inter-connectivity between proteins within a predicted complex, like density measure of a subgraph of a protein-protein interaction (PPI) network. Under such a scoring scheme, the number of proteins within a predicted cluster should be high to some degree, at least three or four, in order that such a score function works effectively. As a result, heterodimeric complexes are left out of consideration.

This weakness of existing tools toward heterodimeric protein complexes implies the need to develop another approach for predicting heterodimeric complexes. In this paper, we present a method for learning naïve Bayes classifiers for heterodimeric complexes. Those classifiers exploit the features for heterodimeric protein complexes which are designed with genome-wide data sets, including PPI data, gene ontology annotations, and protein localization data. Those features are trained by positive examples, a part of known heterodimeric protein complexes, and negative examples, which will be pairs of two proteins that are not known to form heterodimeric protein complexes. Such a feature can be considered to be essentially a pair of conditional probability distributions of possible values of features. One is a distribution of feature values specialized for positive examples, and the other is specialized for negative examples. The log-likelihood ratio by the naïve Bayes classifier with those trained features can be used to score a given pair of two proteins and predict whether the pair forms a heterodimeric protein complexes or not.

It should be noted here that the problem of identification of two components of heterodimeric protein complexes is different from that of identification of PPIs. In the latter problem, it does not matter whether an interaction forms a protein complex with interactions neighboring with that interaction. On the other hand, in the former problem, it is required to determine whether an interaction solely forms a protein complex or not.

We have carried out a five-fold cross-validation over the positive and negative examples derived from known heterodimeric complexes in CYC2008 and known PPIs in WI-PHI, which is a database of PPIs of yeast. This computational experiment shows acceptable performance and gives us interesting insights into heterodimeric protein complexes. Furthermore, the trained classifiers are evaluated by predicting whether each of the PPIs in WI-PHI is heterodimeric or not. It then turns out that those classifiers show higher predictability than various existing algorithms on yeast data sets with the exact matching criterion. Similar results are also obtained with an approximate matching criterion.

## Methods

In this section, we describe our method for predicting heterodimeric protein complexes by naïve Bayes classifiers. For this task, we have designed several features for heterodimeric protein complexes based on a weighted PPI network, semantic similarities for molecular function and biological process aspects of the Gene Ontology, and protein localization information. Here a feature means a (mathematical) function mapping from a pair of proteins to a real number or an integer, which is called a score.

**Figure 1:**
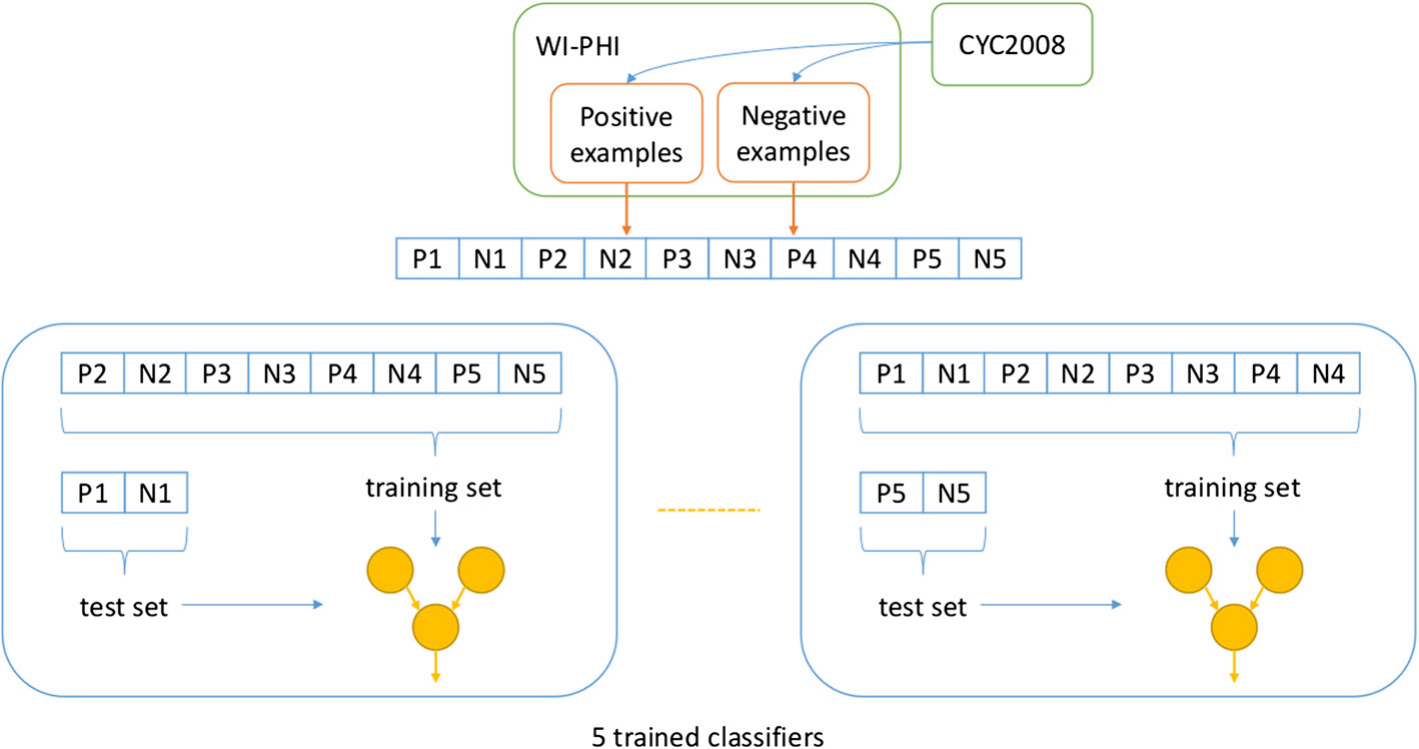
**Overview of a five-fold cross-validation.** This figure gives an overview of the five-fold cross-validation carried out in this work. The positive and negative examples are determined from the WI-PHI and CYC2008 databases.

**Figure 2:**
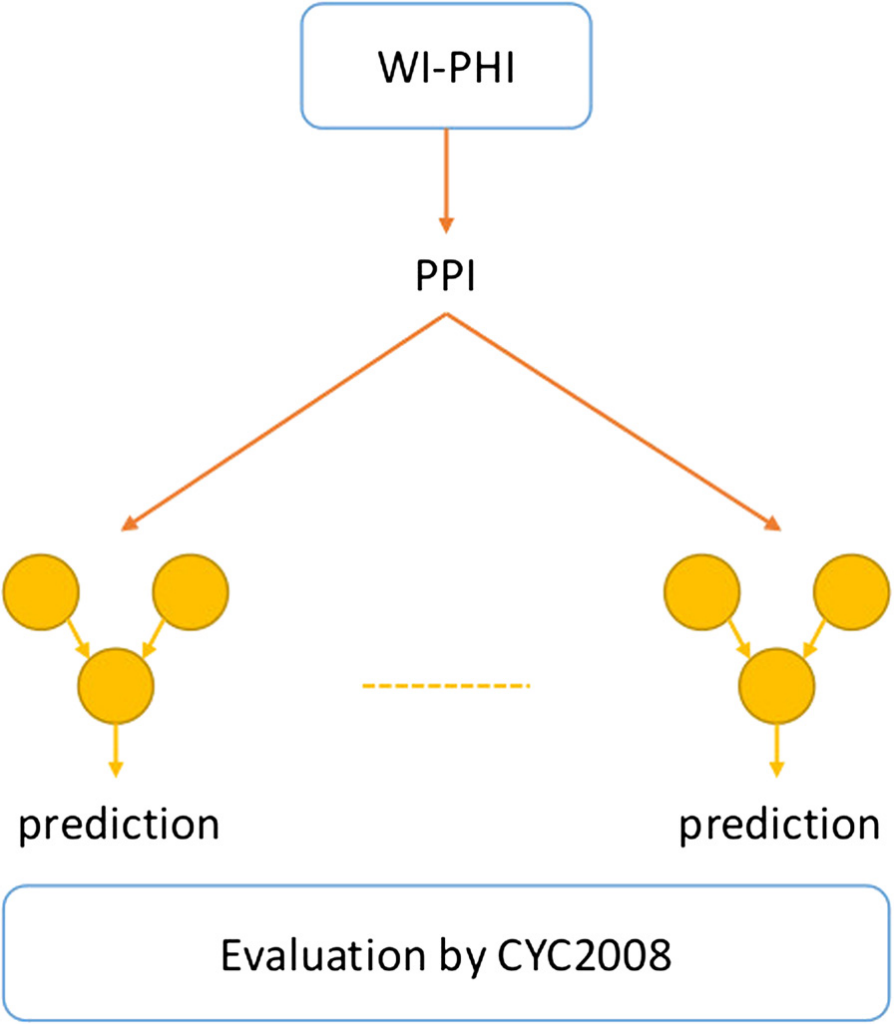
**Overview of evaluation of trained classifiers by all known PPIs.** This figure shows an overview of evaluation of the classifiers trained in a cross-validation by all PPIs in the WI-PHI database.

Given a set of features, a five-fold cross-validation is carried out, in which classifiers are trained with training sets of positive and negative examples and those trained classifiers are evaluated with test sets of positive and negative examples (see Figure 1). The trained classifiers are then used to predict whether each of known PPIs form a heterodimeric protein complex or not (see Figure 2), and the resulting performance is compared with those of other methods.

In the subsequent subsections, we introduce templates for features as well as individual features for a heterodimeric protein complex, and describe details of other parts of our methods.

### Design of features for heterodimeric protein complexes

We here design several features for heterodimeric protein complexes, which will be exploited in a naïve Bayes classifier.

In general, measures of internal connectivity for a subgraph, like density measure, are often used as a feature characterizing heteromeric protein complexes. For example, MCODE [11] is designed based on the observation that densely connected subgraphs may represent known complexes. However, such measures do not work well for heterodimeric protein complexes because the possible states of internal connectivity of a pair of proteins is binary, i.e., connected or not. In general, density-based measures works better for larger complexes. Therefore, we have designed features specialized for heterodimeric protein complexes, which are derived from PPIs, gene ontology annotations, and protein localization data.

Here we introduce three templates for features for heterodimeric protein complexes. Let *e* be a pair of proteins. The combination of a template and a score function for *e* leads to a concrete feature. In this work, four score functions for *e* are formulated based on the following four genome-wide data sets, respectively: (i) PPI weights of WI-PHI [18], (ii) proximity from a protein to another obtained by random walks with restarts on the PPI network derived from WI-PHI, and (iii) semantic similarity for biological process aspect of GO, and (iv) semantic similarity for molecular function aspect of GO, respectively.

Before describing those data sets, a PPI network is introduced as an underlying graph for features. Let *G*=(*V*,*E*) be an undirected graph representing a PPI network where a node is a protein and an edge corresponds to an interaction between the corresponding proteins. This graph is used as the underlying graph for features to be defined here.

For an edge, *e*, let

$$N_{e}=\{e^{\prime }\in E||e^{\prime }\cap e|=1\},$$

representing the edges adjacent to either end point of *e*. This graph, *G*, is made from the WI-PHI database in this work.

WI-PHI [18] is a PPI database with 5955 yeast proteins and 50000 interactions. Among them, 49607 with 5953 proteins are non-self interactions. Each interaction has a weight, which is determined from various heterogeneous data sources, including results of tandem affinity purification coupled to MS (TAP-MS), large-scale yeast two-hybrid studies, and small-scale experiments stored in dedicated databases. The higher the weight is the more reliable it is. The lowest and highest values are 6.6 and 146.6, respectively. If *e* is not included in WI-PHI, the weight of *e* is set to be zero. Hereafter, the weight of *e* is denoted by PPIWeight(*e*).

The next score function of *e* is a proximity of the two nodes between *e* derived from a random walk with restarts [7,8,19-21]. The output of a random walk with restarts at a node, *u*, gives the stationary probability from *u* to the other nodes, *v*, denoted by *π*(*u*→*v*), satisfying the following equation:

$$x_{u}=(1-\alpha )Ax_{u}+\alpha b_{u}$$

where **x**_*u*_=(*π*(*u*→*v*_1_)*π*(*u*→*v*_2_)⋯*π*(*u*→*v*_*n*_))^T^, **b**_*u*_ is the vector whose elements are all 0 except the *u*-th element being 1, *A* is the column-normalized transition matrix derived from *G*, and *α* is the restart probability of a random walk with restarts. Here, *α* is set to be 0.6, the default value of the restart probability of NWE [8]. The different values of 0.3 and 0.9 are also applied, and very similar results are obtained in the cross-validation (data not shown). Since the stationary probabilities between *u* to *v* are not symmetric, namely, *π*(*u*→*v*)≠*π*(*v*→*u*), we here define the symmetric proximity of a random walk with restarts between *u* and *v* as

RandomWalkProximity(u,v)=(π(u→v)+π(v→u))/2.

This score function is also given to templates to generate concrete features.

The Gene Ontology project, or GO, provides a controlled vocabulary to describe gene and gene product attributes in any organism and the GO database is a comprehensive knowledge structures relating functions of genes and their products [22]. Although it is still on-going project, it has already been proved to be effective in the evaluation of human PPIs [23]. Let *X* be an ontology among the two ontologies, Biological Process (BP) and Molecular Function (MF). Here, Cellular Component is excluded because this ontology contains many terms representing subunits or memberships of protein complexes. Yang *et al.* proposed a method for improving existing GO semantic similarity measures in [24]. In this work, the proposed measure based on Lin (the option 38 in their MATLAB tool) is adopted. We denote by SemanticSim.X(*e*) the semantic similarity score of *e* over *X*, which is also used as a score function by the templates. An ontology file shows GO terms and their relationships. The file we used is dated Nov. 21, 2012. and downloaded from http://www.geneontology.org/GO.downloads.shtml. An annotation file contains associations between gene products and GO terms. The file we used is compiled by *Saccharomyces* Genome Database (SGD) [25] and dated Nov. 17, 2012.

Suppose that for a pair of proteins, *e*∈*V*^2^, a score function, *m*, gives a real number, *m*(*e*), which will be one of the followings: PPIWeight(*e*), RandomWalkProximity(*e*), SemanticSim.BP(*e*), or SemanticSim.MF(*e*).

#### Score-type feature

**Figure 3:**
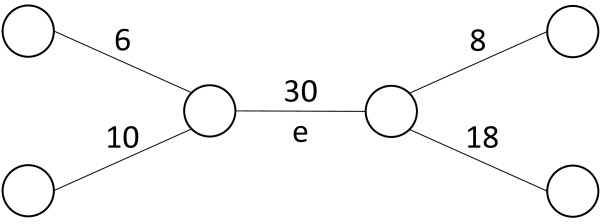
**An example of a subgraph of a PPI network.** This graph consists of an edge, *e*, and its adjacent edges with their weights.

The first template is used to generate a score-type feature. The score-type feature with *m*, denoted by m.Score(*e*), is a function to just return the score of *m* for *e*, *i.e.,m*(*e*). Thus this is the most simplest feature. For example, the returned value is 30 for *e* in the graph in Figure 3. This type features will work well if *m* itself is a good characterization of heterodimeric protein complexes.

#### DiffToMax-type feature

The second template provides a DiffToMax-type feature, which calculates the differences between *m*(*e*) and the maximum of scores of the adjacent edges to *e*, i.e.,

$$\mathrm{m.DiffToMax}(e)=m(e)-\underset{e^{\prime }\in N_{e}}{\text{max}}m(e^{\prime }).$$

For this feature, it is expected that the higher the gap is, the likelier *e* corresponds to a heterodimeric complex. For the edge, *e*, in the graph in Figure 3, we have m.DiffToMax(*e*)=12.

#### Rank-type feature

The last template gives a rank-type feature, denoted by m.Rank(*e*), which returns the number of the adjacent edges to *e* whose scores of *m* are greater than *m*(*e*), i.e.,

$$\mathrm{m.Rank}(e)=|\{e^{\prime }\in N_{e}|m(e^{\prime })>m(e)\}|.$$

For the edge, *e*, in the graph in Figure 3, we have m.Rank(*e*)=0. It can be expected that the higher the returned value to *e* is, the lower the likelihood that *e* is a heterodimeric protein complex.

In addition to the three feature templates, we have designed three individual features as follows.

#### Feature based on protein localization

The next feature is designed based on the observation that two proteins should express in the same location if they interact with each other. Huh *et al.*[26] classified 75% of the yeast proteins into 22 distinct subcellular localization categories by their GFP(green fluorescent protein)-tagged library. By exploiting this localization information, the feature, Localization(*e*), is defined as follows:

$$\begin{array}{llll} \text{Localization}(e)=\left\{\begin{array}{ll} \;1 & \text{if both proteins of}e\text{share at least one category at which they express,}\\ \;0 & \text{if either protein of }e\text{completely has no categories at which it expresses,}\\ -1 & \text{otherwise} \end{array}\right) & & & \end{array}$$

#### Feature based on neighboring common nodes

Existence of nodes neighboring to both nodes, *u* and *v* of *e*, is often used as an index to determine whether *u* and *v* belong to the same protein complex of size three or more (see, for example, [27]). Thus, the number of such nodes can be used as an inverse indicator of *e* being a heterodimeric protein complex. The set of those nodes is equal to *N*_*u*_∩*N*_*v*_, where *N*_*u*_={*z*∈*V*|{*u*,*z*}∈*E*}. Thus, the feature, NeighboringCommonNode(*e*), is defined as

$$\text{NeighboringCommonNode}(e)=|N_{u}\cap N_{v}|.$$

#### Feature based on neighboring edges

The feature, NeighboringEdge(*e*) calculates the number of neighboring edges to *e*, i.e.,

$$\begin{array}{lcrr} \text{NeighboringEdge}(e)=|N_{e}|. & & & \end{array}$$

If *e* corresponds to a heterodimeric protein complex, this feature can be expected to return a low value.

### Positive and negative examples of heterodimeric protein complexes

Positive and negative examples are required in supervised-learning processes. In the problem of heterodimeric protein complex prediction, those examples can be modeled as a pair of different proteins. In this work, a positive example is a pair of proteins satisfying the following conditions: (i) it corresponds to a heterodimeric protein complex in CYC2008, (ii) it is not a proper subset of any other complex in CYC2008, and (iii) it corresponds to a PPI in WI-PHI. This means that positive examples used in the learning process are highly reliable. The total number of the resulting positive examples is 152. On the other hand, negative examples are randomly chosen from PPIs in WI-PHI except all PPIs corresponding to heterodimeric protein complexes in CYC2008. The number of them is set to 1520, ten times that of positive examples. Note that these positive and negative examples are used only in a five-fold cross-validation. After that process, the resulting classifiers are evaluated with all PPIs in WI-PHI.

It should be noted here that some true positive examples would be missed due to the incompleteness of used databases of protein complexes. In addition, some of the current negative examples can be false negative ones (i.e. true heterodimeric protein complexes) due to the same reason. This kind of issues will be resolved by further accumulation and annotation of data.

### Discretization

Maximum likelihood estimation is applied to learn two conditional distributions of each of specified features from a training data set. If a feature returns a real number, the range is discretized into 10 equal-width bins over the interval from the minimum to the maximum among the values of all the positive and negative training examples. If a feature returns an integer, 10 bins are also prepared. For example, in a rank-type feature, the first 9 bins correspond to the feature values, 0,1,⋯,8, respectively, and the last one covers 9 and more. In both cases, the resulting distributions are multinomial distributions. A Dirichlet Prior is applied to avoid the probability being zero of a particular bin. The pseudocount of one is given to each of the bins in this study.

### Naïve Bayes classifier

**Figure 4:**
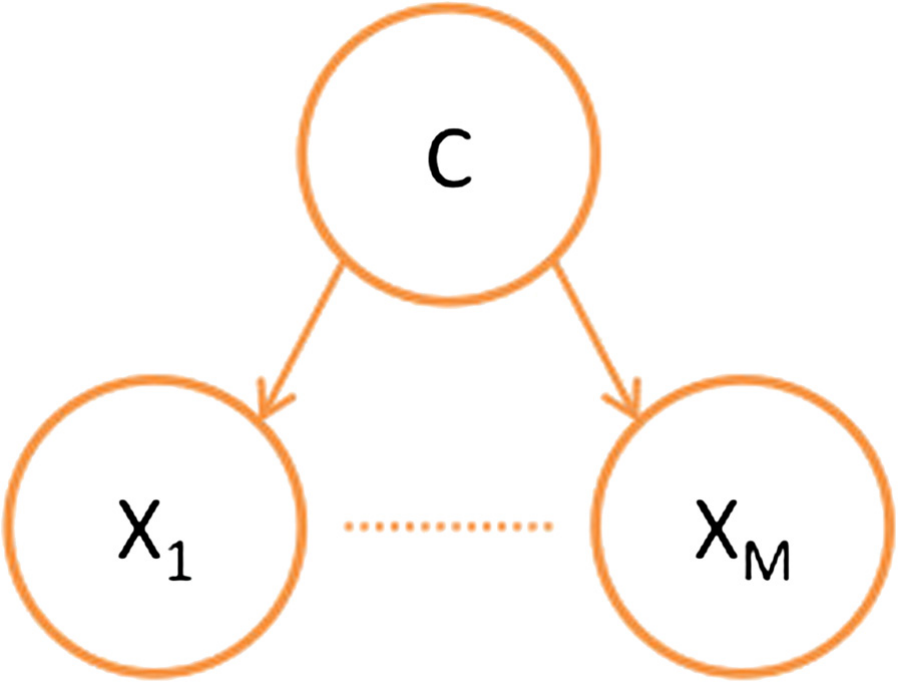
**Naïve Bayesian probabilistic model for scoring a pair of proteins.** The root node ‘C’ is the binary indicator for heterodimeric protein complexes (1 (true) if the pair of proteins forms a heterodimeric protein complex and 0 (false) otherwise). Each of the remaining nodes, labeled *X*_1_,*X*_2_,…,*X*_*M*_, represents a particular features, like ones described in Section ‘Design of features for heterodimeric protein complexes’.

In order to predict heterodimeric protein complexes, we exploit a naïve Bayes classifier, which is a simple probabilistic model based on Bayes’ theorem. Figure 4 presents an overview framework of a naïve Bayes classifier. Let *X*_1_,*X*_2_,…,*X*_*M*_ be random variables for *M* features, and *C* a random variable representing a class whose value is either 1 (true) or 0 (false). In a naïve Bayes classifier, it is assumed that each feature *X*_*j*_ is conditionally independent of every other feature *X*_*k*_ for *k*≠*j*.

For a pair of proteins in a given PPI network, we can compute the conditional probability of how likely it represents a heterodimeric protein complex using the following equation.

$$\begin{array}{ll} P(C|X_{1},X_{2},\ldots ,X_{M}) & =\frac{P(X_{1},X_{2},\ldots ,X_{M}|C)P(C)}{P(X_{1},X_{2},\ldots ,X_{M})}\\ & =\frac{P(C)\overset{M}{\underset{j=1}{\prod }}P(X_{j}|C)}{P(X_{1},X_{2},\ldots ,X_{M})} \end{array}$$

Bayes’ rule is used in the first row of the above equations. The second equation utilizes the conditional independence assumption of the naïve Bayes model to decompose the conditional joint probability to the probabilities of different features.

Let *S* be a pair of proteins to be classified whose feature values are *x*_1_,*x*_2_,…,*x*_*M*_ for *X*_1_,*X*_2_,…,*X*_*M*_, respectively. The log likelihood ratio (LLR) for *S* can be computed using the two posteriors, *P*(*C*=1|*X*_1_=*x*_1_,*X*_2_=*x*_2_,…,*X*_*M*_=*x*_*M*_) and *P*(*C*=0|*X*_1_=*x*_1_,*X*_2_=*x*_2_,…,*X*_*M*_=*x*_*M*_) as follows:

$$\begin{array}{l} \text{LLR}(S)=\log\frac{P(C=1|X_{1}=x_{1},X_{2}=x_{2},\ldots ,X_{M}=x_{M})}{P(C=0|X_{1}=x_{1},X_{2}=x_{2},\ldots ,X_{M}=x_{M})}\\ \;=\log\frac{P(C=1)\overset{M}{\underset{j=1}{\prod }}P(X_{j}=x_{j}|C=1)}{P(C=0)\overset{M}{\underset{j=1}{\prod }}P(X_{j}=x_{j}|C=0)}\\ \;=\log\frac{P(C=1)}{P(C=0)}+\overset{M}{\underset{j=1}{\sum }}\log\frac{P(X_{j}=x_{j}|C=1)}{P(X_{j}=x_{j}|C=0)} \end{array}$$

In order to make two LLRs with the different numbers of features comparable, the above LLR is normalized by dividing by *M*+1. Hereafter LLR means the normalized LLR.

In the learning process of naïve Bayes classifiers, the ratio of $\frac{P(C=1)}{P(C=0)}$ is set to be proportional to the ratio of the number of positive examples to the number of negative examples, which is $\frac{1}{10}$. As a result, the class LLR, $\log\frac{P(C=1)}{P(C=0)}=-2.30$. In the evaluation process of trained classifiers with all PPIs in WI-PHI, the ratio of $\frac{P(C=1)}{P(C=0)}$ is also set to be proportional to the ratio of the number of positive examples to the number of negative examples, which is $\frac{152}{49607-152}=0.0031$. In this case, the class LLR is set to be $\log\frac{P(C=1)}{P(C=0)}=-5.66$.

If the LLR of *S* is greater than a specified threshold, *S* is predicted to be positive, and negative otherwise. The default value of the threshold is set to be 0.6 in order to relatively reduce the number of false positives. Later, we will see how much the value of the threshold affects the predictability. By varying the value of the threshold, a receiver operating characteristic (ROC) curve is obtained.

Here is a remark on functional dependencies between features. It is reported in an empirical study of the naïve Bayes classifier in [28] that even if some features are functionally dependent, naïve Bayes often works well. Thus, in this work, various sets of features which can be functionally dependent are embedded into the same naïve Bayes classifier. Actually, the best performance feature set we have obtained contains features derived from the same source data sets, which will be shown in the result section.

### Performance measures

There have been many unsupervised learning algorithms proposed to predict heteromeric protein complexes, not specialized for heterodimeric complexes. Some of them can predict clusters of size two. Thus, it would be useful to be able to compare performance of *supervised* and *unsupervised* learning algorithms w.r.t. heterodimeric protein complexes. To realize it, firstly, we formulate the three major measures, precision, recall, and F-measure in a general way, which is the same as in [9]. After that, we will explain how to use them.

To formulate those measures, a matching criterion for two sets of proteins is needed. Let *s* and *t* be sets of proteins with arbitrary sizes. The *overlap ratio* between *s* and *t*, denoted by *ov*(*s*,*t*), is defined as follows:

$$\text{ov}(s,t)=\left\{\begin{array}{cc} \frac{\left|s\cap t\right|}{\sqrt{\left|s|\cdot |t\right|}} & \text{if}\;|s\cap t|\geq 2\\ 0 & \text{otherwise}. \end{array}\right)$$

We say that *s* and *t* are matched if *ov*(*s*,*t*) is no less than a predefined threshold, *η*. If *s* and *t* share at least two proteins, the overlap ratio is equal to the ratio of the number of common proteins in *s* and *t* to the geometric mean of the sizes of *s* and *t*. Thus, it is one if *s* and *t* are identical to each other. On the other hand, if *s* and *t* share less than two proteins, the overlap ratio is defined as zero. The reason why the overlap ratio is zero even if *s* and *t* share one protein is to avoid unfavorable situations when the value of *η* is set to be the typical value of 0.4472 ( $=\sqrt{0.2}$) in the literature. Without that criterion, by randomly generating clusters of size two, known complexes of size two can be matched with some of the clusters by chance.

Let *C* be a set of predicted clusters of proteins by an algorithm, and *K* a set of known complexes. We denote by *N*_*pc*_(*C*,*K*,*η*) the number of predicted clusters matched with at least one known complex, i.e.,

$$N_{\text{pc}}(C,K,\eta )=|\{c|c\in C,\exists k\in K,\text{ov}(c,k)\geq \eta \}|,$$

and by *N*_*kc*_(*C*,*K*,*η*) the number of known complexes matched with at least one predicted cluster, i.e.,

$$N_{\text{kc}}(C,K,\eta )=|\{k|k\in K,\exists c\in C,\text{ov}(k,c)\geq \eta \}|.$$

We then define the *precision* of *C* to *K* with *η* as

$$\text{precision}(C,K,\eta )=\frac{N_{\text{pc}}(C,K,\eta )}{\left|C\right|}.$$

In a similar way, the *recall* of *C* to *K* with *η* is defined as

$$\text{recall}(C,K,\eta )=\frac{N_{\text{kc}}(C,K,\eta )}{\left|K\right|}.$$

The *F-measure* of *C* to *K* with *η* is defined as the harmonic mean of the corresponding precision and recall. Namely, we have

$$F(C,K,\eta )=2\cdot \frac{\text{precision}(C,K,\eta )\cdot \text{recall}(C,K,\eta )}{\text{precision}(C,K,\eta )+\text{recall}(C,K,\eta )}.$$

In this work, two different matching criteria, which are exact and approximate ones, respectively, are used to evaluate predicted clusters of size two. For the set of all clusters predicted by an algorithm, *C*, we denote by *C*|_2_ the subset of all clusters of size two in *C*. Notice that *C* is equal to *C*|_2_ if *C* is generated by our classifiers. Let *K* be the set of all known complexes in CYC2008, and *K*|_2_ the set of all known heterodimeric complexes in CYC2008. The precision and recall with the exact matching criterion for size two are given as *precision*(*C*|_2_,*K*|_2_,1) and *recall*(*C*|_2_,*K*|_2_,1), respectively. Note that all the clusters and complexes used in the measures are of size two. The precision and recall with the approximate matching criterion with *η* for size two are given as *precision*(*C*|_2_,*K*,*η*) and *recall*(*C*,*K*|_2_,*η*), respectively, where *η* is set to be $\eta =\sqrt{0.2}$, the typical value in the literature.

### K-L divergence

The Kullback-Leibler (K-L) divergence of two trained conditional distributions of a feature can be used as a measure for indicating how discriminative the feature is. The K-L divergence is a measure of the difference between two probability distributions, and defined as follows [29]:

$$\text{KL}(P||Q)=\underset{i}{\sum }P(i)\underset{2}{\log}\frac{P(i)}{Q(i)}.$$

Note that in general the Kullback-Leibler divergence is not symmetric, namely *KL*(*P*||*Q*)≠*KL*(*Q*||*P*). The symmetric and non-negative Kullback-Leibler divergence is defined as follows:

$${\text{KL}}^{\text{sym}}(P||Q)=\frac{1}{2}\left(\text{KL}(P||Q)+\text{KL}(Q||P)\right).$$

For a feature *X*_*i*_ with two trained conditional distributions, *P*(*X*_*i*_|*C*=1) and *P*(*X*_*i*_|*C*=0), the symmetric K-L divergence of *X*_*i*_ is defined as

$${\text{KL}}^{\text{sym}}(P(X_{i}|C=1)||P(X_{i}|C=0)).$$

Hereafter the symmetric K-L divergence is simply called the K-L divergence.

## Results and discussion

### Finding the best feature set

**Figure 5:**
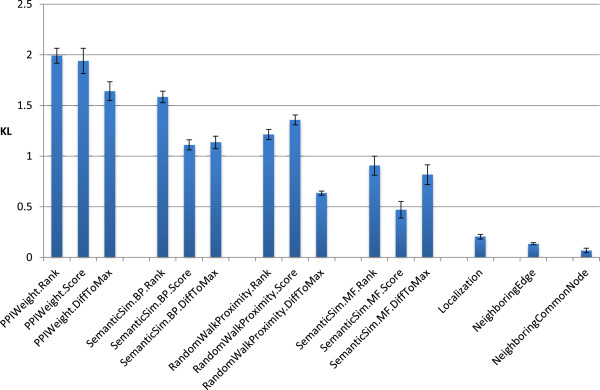
**K-L divergences of features.** The K-L divergence of each feature is calculated with five training sets of positive and negative examples. The mean and standard deviation of five divergence values of a feature are represented by the height of the corresponding bar and the error bar on the top, respectively.

In the first stage of a five-fold cross-validation, the K-L divergence of the 15 designed features is calculated. The mean with standard deviation of those values of each feature over the five folds is shown in Figure 5. The following observations are obtained from Figure 5. At first, the K-L divergences of the three features, Localization, NeighboringEdge, and NeighboringCommonNode are relatively considerably lower than the others. Their K-L divergences are 0.2 or less. On the other hand, those of the other features are about 0.5 or more. As a result, the three features seem to be relatively less effective to predict heterodimeric protein complexes. Thus, in the next stage, these three features are excluded. Secondly, the highest K-L divergence of 2.0 is given by PPIWeight.Rank. Thus this feature is expected to be the most effective feature to discriminate heterodimeric protein complexes from the other complexes. Lastly, the K-L divergence of a feature derived from a feature template is largely dependent on the score function embedded in the feature. When considering the K-L divergence averaged over the different templates with the same score function, those score functions are sorted as follows: PPIWeight, SemanticSim.BP, RandomWalkProximity, and SemanticSim.MF. Thus, according to this order, the corresponding features are expected to contribute the discrimination of heterodimeric protein complexes from the others.

In the second stage of finding discriminative naïve Bayes classifiers, an exhaustive search was executed in the following search space for feature sets. At first, any of the three low K-L divergence features are not included in all the feature sets in the search space. Secondly, for each of the four score function for a pair of proteins, PPIWeight, SemanticSim.BP, RandomWalkProximity, and SemanticSim.MF, three concrete features, Rank, Score, and DiffToMax, are created. Lastly, any feature set in the search space should include one or two features from the four concrete features derived from the same score function. Thus, the total number of feature sets in the search space is 1296.

For each of the feature sets in the search space, a five-fold cross-validation was carried out. Note that the predictability of trained classifiers are evaluated with five test sets of the cross-validation. In the next section, the feature set with the highest mean of F-measures on the five test sets is analyzed deeply.

### Analyses

The best feature set found in the previous section consists of the followings: PPIWeight.Score, PPIWeight. DiffToMax, SemanticSim.BP.Rank, SemanticSim.BP.Diff ToMax, RandomWalkProximity.Rank, RandomWalkProximity.Score, and SemanticSim.MF.DiffToMax. It is interesting that PPIWeight.Rank, whose K-L divergence is the highest among the 15 features, is not included in the above feature set. Instead of that, PPIWeight.Score and PPIWeight.DiffToMax are contained. Probably, the combination of them would work better than PPIWeight.Rank alone. The results on test sets in the five-fold cross-validation are shown in Table 1. Recall that the numbers of positive and negative examples are 152 and 1520, respectively. Each of them is predicted by one of the five trained classifiers to be either positive or negative in the cross-validation. Totally, 98 (64.5%) of the 152 positive examples are correctly predicted to be positive. Namely, they are true positive, and the 54 remaining positive examples are false negatives. On the other hand, 1498 (98.6%) of 1520 negative examples are true negative and the other 22 ones are false positives.

**Table 1:** Prediction result on test sets in five-fold cross-validation

**Fold**	**1**	**2**	**3**	**4**	**5**	**Total**
TP	15	22	25	18	18	98
FN	16	9	5	12	12	54
TN	299	299	298	300	302	1498
FP	5	5	6	4	2	22

This table shows the numbers of true positives (TP), false negatives (FN), true negatives (TN), and false positives (FP) for the test set of each fold. Note that the number of positive examples is 31 in the first and second folds, and 30 in the other folds. The number of negative examples is 304 in each fold.

**Figure 6:**
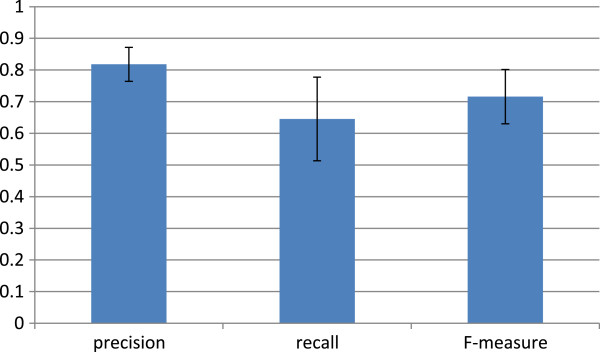
**Performance of trained classifiers by test sets.** This graph shows the mean and standard deviation of precision, recall, and F-measure of the five trained classifiers. These scores are derived from five test sets of positive and negative examples.

For each test set, the performance measures of precision, recall, and F-measure are calculated. Their means with standard deviations are shown in Figure 6. The precision, recall, and F-measure are averagely 0.818, 0.645, and 0.716, respectively. These scores are acceptable result. This feature set is also applied to several different sets of training and test, and quite similar results are obtained (data not shown).

It is interesting to see how many classes predicted by the five trained classifiers are consistent for each of the 152 positive and 1520 negative examples. Table 2 shows the frequency of positive and negative examples according to the number of correct predictions by the classifiers. It can be found that 97 (63.8%) of the positive examples are consistently predicted correctly by the five trained classifiers. In the next section, two instances are examined. Interestingly, there are many 48 (31.6%) positive examples that are consistently predicted to be negative by the classifiers. One of them is also picked up and the reason for the misclassification is considered. Notice that, totally, 145 (95.4%) of the positive examples are predicted to be the same class by the five trained classifiers. Furthermore, 1494 (98.3%) of the negative examples are consistently predicted to be negative by the classifiers, and 14 (0.9%) negative examples are consistently predicted to be positive. Totally, 1508 (99.2%) of the negative examples are consistently predicted by the classifiers. As a result, the five trained classifiers are fairly consistent to each other.

**Table 2:** Consistency of predicted classes

	**0**	**1**	**2**	**3**	**4**	**5**
P	48	4	0	3	0	97
N	14	5	2	2	3	1494

This table shows the frequency of positive and negative examples according to the number of correct predictions by the five trained classifiers. The first row shows the number of classifiers whose predicted classes are correct. The next rows, P and N, give the results on positive and negative examples, respectively.

In the subsequent sections, some of true positives, false negatives, and false positives, which are consistently predicted by the five trained classifiers, are analyzed further.

#### True positive

**Figure 7:**
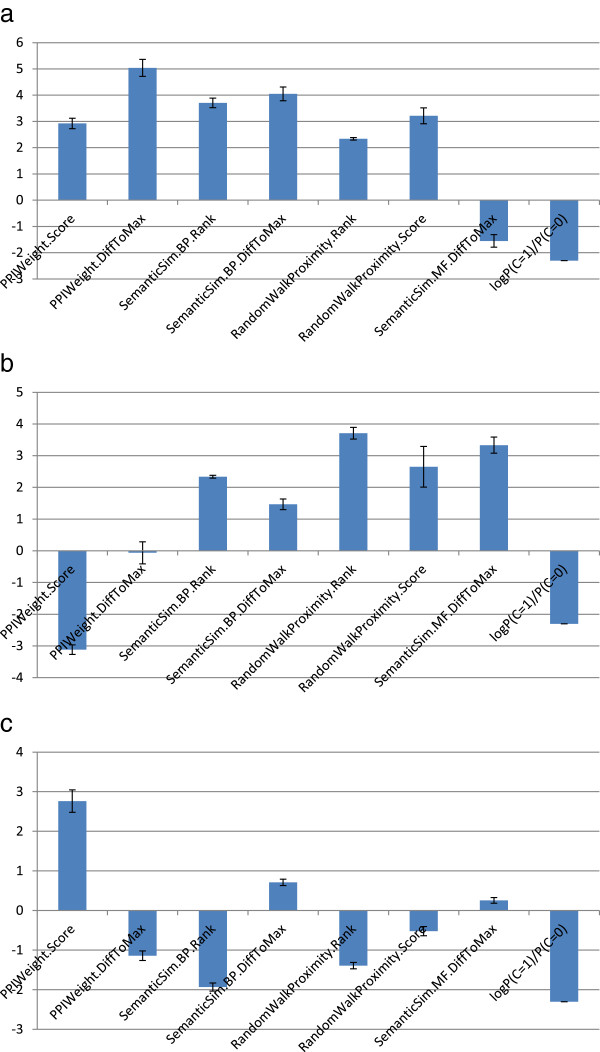
**LLRs of features of positive examples.** These graphs show the LLRs of features for particular instances. Those are **(a)** UBP3/YER151C and BRE5/YNR051C, **(b)** ECM17/YJR137C and MET10/YFR030W, and **(c)** CDC28/YBR160W and CLN1/YMR199W.

The first instance of true positives is the pair of two proteins, UBP3/YER151C and BRE5/YNR051C. These proteins are known to interact with each other to co-regulates anterograde and retrograde transport between the endoplasmic reticulum and Golgi compartments [30]. There is no other known complexes including at least one of the two proteins in CYC2008. The scores of the features to this instance are given in the column of TP1 of Table 3. The PPI weight of the pair is shown to be 79.5, which is relatively high. This means that feature PPIWeight.Score gives a high score to the pair. In addition, UBP3/YER151C and BRE5/YNR051C have 30 and 8 interactions in WI-PHI, and none of the neighboring interactions to the two proteins, except the pair, has a PPI weight higher than that of the pair, 79.5. This means that PPIWeight.DiffToMax, RandomWalkProximity.Rank, and RandomWalkProximity.Score, can return high scores to this pair. Actually, RandomWalkProximity.Rank marks the optimal score of 0 (see Table 3). In addition, this pair has statistically significant biological process GO term, “ribophagy” with p-value 9.58e-06, indicating SemanticSim.BP features return high scores to this pair. Notice that SemanticSim.BP.Rank marks the optimal score of 0. Figure 7 (a) shows the mean of LLRs of each feature, *X*_*j*_, by the five trained classifiers.

**Table 3:** Raw scores of features

**Feature**	**TP1**	**TP2**	**FN**
PPIWeight.Score	79.5	12.8	58.3
PPIWeight.DiffToMax	39.5	-7.2	-47.4
RandomWalkProximity.Rank	0	0	9
RandomWalkProximity.Score	0.0953	0.0509	0.0231
SemanticSim.BP.Rank	0	0	9
SemanticSim.BP.DiffToMax	0.728	0.269	-0.808
SemanticSim.MF.DiffToMax	-2.00	1.22	-1.08

This table shows the returned values of features to particular examples. The columns of TP1, TP2, and FN correspond to the pairs of UBP3/YER151C and BRE5/YNR051C, ECM17/YJR137C and MET10/YFR030W, and CDC28/YBR160W and CLN1/YMR199W, respectively.

All the features, except SemanticSim.MF.DiffToMax, have scores greater than two. As a result, all the whole LLRs obtained by the five trained classifiers are more than 2.0, respectively, which is higher than the threshold, 0.6.

This first instance can be trivial because the PPI weight of the pair is high. This means high LLRs of features derived from the PPIWeight score function. We then pick up all positive examples whose corresponding PPI weights are at most 20 and which are consistently predicted to be positive by the five trained classifiers. The following 15 instances satisfy this criteria: YPL147W YKL188C; YNL246W YLL002W; YOR363C YAL051W; YCL009C YMR108W; YJR135W-A YGR181W; YJR137C YFR030W; YBR036C YBR161W; YFL041W YBR207W; YDL099W YDR517W; YLR067C YJL209W; YIR021W Q0115; YHR079C-A YPL121C; YCL017C YER048W-A; YJR035W YKL054C; YOR321W YDL093W. Among them, the pair of ECM17/YJR137C and MET10/YFR030W is taken as the second instance to be analyzed.

The protein complex formed by those proteins is known as sulfite reductase complex (NADPH). CYC2008 does not contain any other complexes including one of the proteins. The scores and LLRs of the features to this instance are given in the column of TP2 of Table 3 and Figure 7 (b), respectively. This pair’s PPI weight is 12.8, which is considerably low. As a result, the LLR by PPIWeight.Score is averagely below -3 (See Figure 7 (b)). ECM17/YJR137C has four PPIs. The highest PPI is one with MET10/YFR030W. On the other hand, MET10/YFR030W has 48 PPIs. The number of PPIs whose weights are higher than the PPI weight with ECM17/YJR137C is only four. Thus, the scores of RandomWalkProximity features for this pair are relatively high because most of the neighboring PPIs to the pair have lower weights than that of the pair, although the PPI weight of the pair is absolutely low. Actually, the LLR of RandomWalkProximity.Rank is the highest among all the seven features (see Figure 7 (b)). In addition, the most statistically significant biological process GO terms are hydrogen sulfide metabolic process and hydrogen sulfide biosynthetic process with p-value 2.33e-05. Furthermore, the most statistically significant molecular function GO term is sulfite reductase (NADPH) activity with p-value 2.33e-07. As a result, the LLRs of SemanticSim features are high. Thus, in this case, the fault of PPIWeight features is covered by the other features, so that this example is correctly predicted to be positive.

#### False negative

In this section, an instance is picked up from the 48 (31.6%) positive examples that are consistently predicted to be negative by the five trained classifiers, and examined, too.

The pair of CDC28/YBR160W and CLN1/YMR199W is known to form Cdc28p/Cln1p complex, which is a cyclin-dependent kinase complex to promote the G1 to S phase transition [31]. Especially, CDC28/YBR160W is well known as a master regulator of mitotic and meiotic cell cycles and to form nine heterodimeric cyclin-dependent kinase complexes with CLB1, CLB2, ⋯, CLB6, CLN1, CLN2, and CLN3, respectively. Thus, this protein can be considered to be a hub protein. On the other hand, no other complexes including CLN1/YMR199W are known. The scores and LLRs of the features to this instance are given in the column of FN of Table 3 and Figure 7 (c), respectively. The PPI weight of the pair is 58.3. Thus, The LLR of PPIWeight.Score is high. However, the others’ LLRs are weak as follows. CDC28/YBR160W has 75 PPIs, and three of them have higher weights than that of the pair. The highest is 105.7 given with YBR135W. As a result, PPIWeight.DiffToMax gives a negative LLR. CLN1/YMR199W has 26 PPIs, and none of them have higher weights than that of the pair. Although the PPI weight of the pair is relatively high, the pair has many neighboring PPIs. This would make the LLRs of the RandomWalkProximity features weak. To make matters worse, the pair has no statistically significant GO terms in both biological process and molecular function aspects. As a result, this pair is incorrectly predicted. As long as a component of a heterodimeric complex is a hub protein, it might be difficult to detect the complex correctly even if appropriate GO terms were assigned to the two proteins of the complex.

#### False positive

Lastly, we discuss false positives. Among them, two interesting cases can be found. One is the case where either or both proteins of a given pair of proteins are components of a known heteromeric protein complex of size three or more. This result indicates that the features used here are still not enough to discriminate heterodimeric protein complexes from heteromeric ones. Note that, among the 14 negative examples that are consistently predicted to be positive, nine negative examples are in this group, which can be identified in the section of the results on negative examples in Additional file 1. Another case is that a pair of proteins can be a true heterodimeric complex. Actually, among the five remaining false positives, the pair of GIR2/YDR152W and RBG2/YGR173W is known to be a heterodimeric protein complex [31].

In addition, MSH4/YFL003C and MSH5/YDL154W are known to form a dimer with each other. These pairs can be also positive examples. Notice that the set of negative examples used in the cross-validation corresponds to only 3% of the PPIs of WI-PHI. Among them, false positives with high LLRs could be good candidates for positive examples.

#### ROC curve

**Figure 8:**
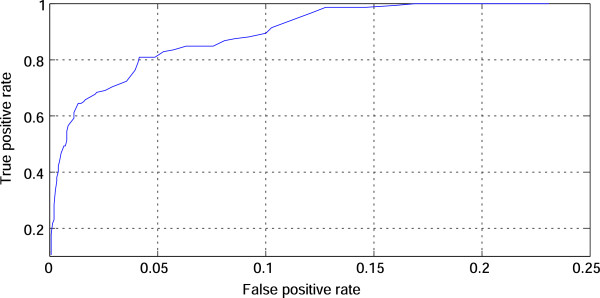
**ROC curve.** This graph shows a ROC curve, which is obtained by changing the LLR threshold from -1 to 1.95 with 0.05 increments in between

A ROC curve is given in Figure 8. This is obtained from the results whose LLR thresholds are ranged from -1 to 1.95 with 0.05 increments in between. Let tp_*i*_,fn_*i*_,tn_*i*_, and fp_*i*_ be the numbers of true positives, false negatives, true negatives, and false positives, respectively, in the *i*-th fold of the five-fold cross-validation. Next, TP is defined as the sum of tp_*i*_ for *i*=1,2,…,5. This is equivalent to the total number of true positives in the cross-validation. In the same way, FN,TN, and FP are given. The false positive rate and true positive rate are given as FP/(FP+TN) and TP/(TP+FN). The ROC curve is created by plotting the false positive rate vs. the true positive rate at each LLR threshold. We can see that as the false positive rate increases, the true positive rate quickly becomes large and saturated. Actually, for the false positive rate of 0.169, the corresponding true positive rate is 1. The area under the ROC curve (AUC) is 0.974. Thus, we can conclude that our method is successful in the cross-validation. Note that the peak of F-measure is given by the LLR threshold ranging from 0.0 to 0.7. The threshold of 0.1 gives the best of averaged F-measure of 0.729 with the standard deviation 0.037. The corresponding precision and recall are 0.670 with 0.077 and 0.809 with 0.071, respectively.

#### Raw output data

Additional files 1 and 2 provide raw outputs of our method for the feature set analyzed in this section. Additional file 1 is the main output file of our tool. Additional file 2 gives the pair of trained multinomial distributions of a feature for positive and negative examples in each fold of the five-fold cross-validation.

### Performance comparison

Qi *et al.*[32] have proposed a supervised approach with a Bayesian classifier for protein complex prediction. However, their target is complexes composed of *three* or more proteins. Most of the features embedded into their classifier are specialized to work well for relatively large complexes. Thus it will be difficult to apply their method to heterodimeric protein complex prediction.

On the other hand, there have been many *unsupervised* learning methods proposed to predict *heteromeric* protein complexes. Thus, to see how much performance our method achieves, performance comparison is carried out with the following nine unsupervised protein complex prediction tools, MCL [6], RRW [7], NWE [8], PPSampler [9], RNSC [10], MCODE [11], DPClus [12], CMC [13], and COACH [14]. However, the last four tools are excluded from further analysis because they do not return any predicted clusters of size two. Note that the above tools are all executed with their default settings, except the option of the minimum size of predicted complexes of RRW and NWE, which are set to be two. Recall that features based on functional information, which are based on the biological process and molecular function ontologies, respectively, are exploited by our method. Thus, our method should be compared with another algorithm incorporating functional information in protein complex prediction. RNSC (Restricted Neighborhood Search Clustering Algorithm) [10] is such a method and adopted in this comparison because it is publicly available.

Usually, all PPIs of a database are taken as input to unsupervised learning algorithms. Here, all WI-PHI PPIs are given to the above unsupervised learning algorithms. In order to compare our classifiers with those algorithms as fair as possible, the five trained classifiers are imposed on the prediction of the class of the pair of the proteins of each of the PPIs.

**Figure 9:**
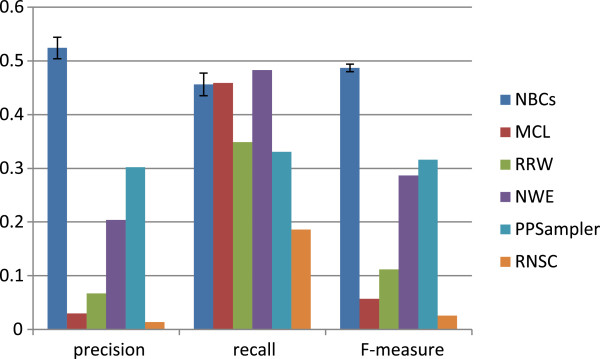
**Performance comparison with the approximate matching criterion.** The five trained classifiers and five unsupervised learning methods are compared in precision, recall, and F-measure, which are determined with the approximate matching criterion with $\eta =\sqrt{0.2}$.

In the literature, heteromeric predicted clusters are *approximately* evaluated whether they are matched with some known complexes. A typical matching threshold is $\eta =\sqrt{0.2}$ (see, for example, [9,11,14,33]). Thus, *precision*(*C*|_2_,*K*,*η*) and *recall*(*C*,*K*|_2_,*η*) are calculated with $\eta =\sqrt{0.2}$. It should be noted here that, when *C* is given by our classifiers, we have *C*|_2_=*C*, *i.e.,recall*(*C*,*K*|_2_,*η*) is equal to *recall*(*C*|_2_,*K*|_2_,*η*). On the other hand, in general, it does not hold for unsupervised learning methods. Thus, this performance comparison with this approximate matching criterion is advantageous to unsupervised learning methods. However, as shown in Figure 9, in F-measure, the classifiers are superior to the unsupervised learning methods (Details are shown soon).

The number of predicted clusters, *N*_*pc*_, and *N*_*kc*_ under $\eta =\sqrt{0.2}$ are shown in Table 4. Those numbers of our method are averaged over the five trained classifiers. It can be seen that the number of predicted clusters varies with the individual tool, ranging from 129 to 1824. Similarly, it holds for *N*_*pc*_ and *N*_*kc*_, respectively. From these numbers, the three performance measures of precision, recall, and F-measure are calculated and the resulting graph is shown in Figure 9. The precision of our classifiers is slightly more than 0.5, followed by 0.3 of PPSampler. The best performer in recall is NWE, closely followed by MCL and our classifiers. These tools form the top group. As a result, the F-measure of our classifiers turns to be the best, which is 0.487±0.007, followed by 0.316 of PPSampler and 0.287 of NWE. Thus it is 54% better than the second.

**Table 4:** **Performance with the approximate matching criterion with**$\eta =\sqrt{0.2}$

**Method**	**NBCs**	**MCL**	**RRW**	**NWE**	**PPSampler**	**RNSC**
#cluster	298±18	213	1824	632	129	576
*N*_*p* *c*_	156±6	7	122	129	39	8
*N*_*k* *c*_	78±4	79	60	83	57	32

The first row shows the name of a prediction algorithm. The second row gives the number of predicted clusters of size two. The subsequent columns show *N*_*pc*_ and *N*_*kc*_ calculated with $\eta =\sqrt{0.2}$. The column of NBCs (naïve Bayes classifiers) gives the result of our method.

**Figure 10:**
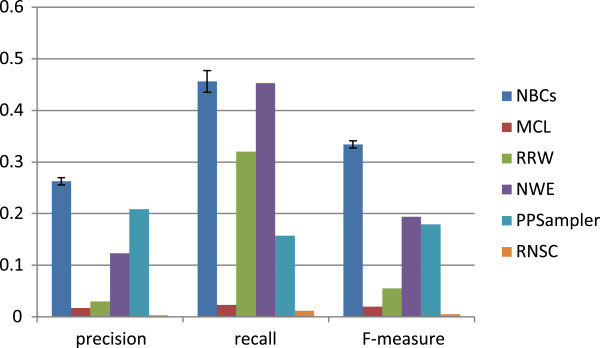
**Performance comparison with the exact matching criterion.** The five trained classifiers and five unsupervised learning methods are compared in precision, recall, and F-measure, which are determined with the exact matching criterion.

Next is the performance comparison with the exact matching criterion of *η*=1. In this case, *N*_*pc*_ is equal to *N*_*kc*_, which is shown in Table 5. It varies from 2 to 78. The resulting precision, recall, and F-measure are shown in Figure 10. In can be seen that the precision of the classifiers is still higher than those of the unsupervised learning methods, although the gap between the best and the second best precision score, 0.209, given by PPSampler, is smaller than that with the approximate matching criterion. In recall, the classifiers and NWE, which are almost the same, are 43% better than the third best score of 0.32, given by RRW. Finally, the best F-measure, 0.334±0.007, is also achieved by the classifiers, followed by NWE and PPSampler whose F-measures are 0.194 and 0.179, respectively. Thus, the best one is 74% and 88% better than them, respectively.

**Table 5:** Performance comparison with the exact matching criterion

**Method**	**NBCs**	**MCL**	**RRW**	**NWE**	**PPSampler**	**RNSC**
*N*_*pc*_(=*N*_*kc*_)	78±4	4	55	78	27	2

The second row shows *N*_*pc*_(=*N*_*kc*_) calculated with *η*=1.

By comparing Figures 9 and 10, the following observations is obtained. At first, the precision of all tools are reduced. This fact indicates that some predicted clusters of size two are approximately matched with strictly larger known complexes, *t*. Note that |*t*| is limited to ten in this work because of $\text{ov}(s,t)\geq \sqrt{0.2}$ with |*s*|=2. Notice that those predicted clusters are completely included in the matching known complexes from the definition of the overlap ratio. Secondly, the recall of all the unsupervised learning methods, especially MCL, PPSampler, and RNSC, are also reduced. This fact indicates that some of the known complexes of size two, *i.e.,* heterodimeric protein complexes, are approximately matched with predicted clusters of size ranging from three to ten. These two observations imply the difficulty of predicting heterodimeric protein complex exactly.

Although the trained classifiers outperforms other methods, the performance measures are lower than those in the cross-validation. One of the reasons is that the unbalanced ratio of the number of negative examples to that of positive ones. The ratio is 49448 to 159. These numbers are obtained as follows. CYC2008 contains 172 heterodimeric protein complexes. Among them, 13 heterodimeric complexes do not have the corresponding PPIs in WI-PHI. Thus, 159 positive examples are determined from WI-PHI and CYC2008. Recall that WI-PHI has 49607 non-self interactions. Thus, the resulting negative examples in the WI-PHI database is 49448. In general, to avoid making many false positives, the LLR threshold and the class LLR, $\log\frac{P(C=1)}{P(C=0)}$ should be relatively low. Actually, the class LLR is set to be lower than in the cross-validation. This causes that the number of true positives, 98, in the cross-validation (Table 1) is reduced to 78±4 in this performance comparison (Table 5). Another reason is due to not yet known PPIs nor heterodimeric protein complexes. Thus, some of the current negative examples, determined from the WI-PHI and CYC2008 databases, can be positive examples, as shown in Section ‘Analyses’. If these data sets are expanded quantitatively and qualitatively, prediction can be more accurate. Lastly, information on PPIs and heterodimeric protein complexes being static is also another reason, because they are intrinsically dynamic cellular entities. If time- and context-dependent PPIs and protein complexes are available, more sophisticated features could discriminate heterodimeric protein complexes from the others more correctly.

### Potential protein complexes

We have conducted further analysis as follows. All pairs of proteins, *x*, satisfying the following conditions are extracted: (i) *x* is known to have an interaction between the proteins of *x*, (ii) *x* does not correspond to any heterodimeric protein complexes, and (iii) *x* is predicted to be positive by all the five classifiers. The total number of those PPIs are 154. Among them, 51 (33%) of them are completely included in known complexes of size three or more. Thus, some of the remaining 103 PPIs are candidates for true heterodimeric complexes. In addition, many of 103 PPIs can be potential subunits of undiscovered protein complexes of size three or more because the fact that they are predicted to be positive by the five classifiers implies that they are functionally and topologically closely related. Thus, these PPIs are good candidates for unknown protein complexes. Raw data of this analysis can be found in the last part of Additional file 1.

### Future works

Currently, there is no high-quality weighted PPI data in human, like WI-PHI in yeast. It is a future work to create such data set and apply our method to human data sets. In addition, it is also an interesting future work to apply classifiers trained by yeast data sets to other organisms. In this case, the requirement is at least input data sets to features embedded into the classifiers.

It is also a future work to design more sophisticated features or templates for concrete features using some genome-wide data sets. Especially, a feature based on 3D structure information can be promising.

Very recently, an independent work for predicting heterodimeric protein complexes by a support vector machine (SVM) with new features based on protein domain information has been published [34]. Although the best F-measure of the proposed method in a ten-fold cross-validation is 0.631, which is lower than 0.716 of our best F-measure in the five-fold cross-validation, it would be worth considering to apply existing kernel functions to the problem and to design new kernel functions. Furthermore, in addition to SVMs, other machine learning classification tools like decision trees and random forests should be considered.

## Conclusions

In this paper, we have proposed a supervised learning method for heterodimeric protein complexes. For this purpose, we have designed templates for features and individual features, which is based on genome-wide data. The naïve Bayes classifiers are evaluated in a five-fold cross-validation, and the trained classifiers are also tested with all known PPIs. Those classifiers are shown to attain much better performance than existing unsupervised learning methods.

## Competing interests

The article processing charge was funded by the Institute of Mathematics for Industry at Kyushu University. There is no other financial or non-financial competing interests.

## Supplementary Material

Additional file 1 **Main output file.** This file is the main output file of our tool, which contains results of the five-fold cross-validation and the evaluation of the five trained classifiers with all PPIs of WI-PHI. Click here for file

Additional file 2 **Parameters of trained naïve Bayes classifiers.** This file shows for a feature, the pair of multinomial distributions trained by positive and negative training examples in each fold of the cross-validation. Click here for file
